# Expression of lymphoid structure-associated cytokine/chemokine gene transcripts in tumor and protein in serum are prognostic of melanoma patient outcomes

**DOI:** 10.3389/fimmu.2023.1171978

**Published:** 2023-06-22

**Authors:** Lilit Karapetyan, Hassan M. AbuShukair, Aofei Li, Andrew Knight, Ayah Nedal Al Bzour, Ian P. MacFawn, Zachary J. Thompson, Ann Chen, Xi Yang, Rebekah Dadey, Arivarasan Karunamurthy, Danielle Vargas De Stefano, Cindy Sander, Sheryl R. Kunning, Yana G. Najjar, Diwakar Davar, Jason J. Luke, William Gooding, Tullia C. Bruno, John M. Kirkwood, Walter J. Storkus

**Affiliations:** ^1^ Department of Cutaneous Oncology, H. Lee Moffitt Cancer Center and Research Institute, Tampa, FL, United States; ^2^ Faculty of Medicine, Jordan University of Science and Technology, Irbid, Jordan; ^3^ Department of Pathology, University of Pittsburgh Medical Center, Pittsburgh, PA, United States; ^4^ Department of Medicine, Division of General Internal Medicine, University of Pittsburgh Medical Center, Pittsburgh, PA, United States; ^5^ Department of Immunology, University of Pittsburgh Medical Center (UPMC) Hillman Cancer Center, Pittsburgh, PA, United States; ^6^ Department of Bioinformatics and Biostatistics, The Moffitt Cancer Center and Research Institute, Tampa, FL, United States; ^7^ Department of Medicine, Brigham and Women’s Hospital and Dana Farber Cancer Institute, Boston, MA, United States; ^8^ Department of Medicine, Hillman Cancer Center, Division of Hematology/Oncology; University of Pittsburgh Medical Center, Pittsburgh, PA, United States; ^9^ Hillman Cancer Center Biostatistics Facility, University of Pittsburgh Medical Center Hillman Cancer Center, Pittsburgh, PA, United States; ^10^ Tumor Microenvironment Center, University of Pittsburgh Medical Center (UPMC) Hillman Cancer Center, Pittsburgh, PA, United States; ^11^ Department of Immunology, University of Pittsburgh School of Medicine, Pittsburgh, PA, United States; ^12^ Departments of Dermatology, Pathology and Bioengineering, University of Pittsburgh Medical Center, Pittsburgh, PA, United States

**Keywords:** tertiary lymphoid structure, lymphoid aggregate, melanoma, APRIL, TNFSF13, cytokine, chemokine, survival

## Abstract

**Background:**

Proinflammatory chemokines/cytokines support development and maturation of tertiary lymphoid structures (TLS) within the tumor microenvironment (TME). In the current study, we sought to investigate the prognostic value of TLS-associated chemokines/cytokines (TLS-kines) expression levels in melanoma patients by performing serum protein and tissue transcriptomic analyses, and to then correlate these data with patients clinicopathological and TME characteristics.

**Methods:**

Levels of TLS-kines in patients’ sera were quantitated using a custom Luminex Multiplex Assay. The Cancer Genomic Atlas melanoma cohort (TCGA-SKCM) and a Moffitt Melanoma cohort were used for tissue transcriptomic analyses. Associations between target analytes and survival outcomes, clinicopathological variables, and correlations between TLS-kines were statistically analyzed.

**Results:**

Serum of 95 patients with melanoma were evaluated; 48 (50%) female, median age of 63, IQR 51-70 years. Serum levels of APRIL/TNFSF13 were positively correlated with levels of both CXCL10 and CXCL13. In multivariate analyses, high levels of serum APRIL/TNFSF13 were associated with improved event-free survival after adjusting for age and stage (HR = 0.64, 95% CI 0.43-0.95; p = 0.03). High expression of *APRIL/TNFSF13* tumor transcripts was significantly associated with improved OS in TCGA-SKCM (HR = 0.69, 95% CI 0.52-0.93; p = 0.01) and in Moffitt Melanoma patients (HR = 0.51, 95% CI: 0.32-0.82; p = 0.006). Further incorporation of *CXCL13 and CXCL10* tumor transcript levels in a 3-gene index revealed that high *APRIL/CXCL10/CXCL13* expression was associated with improved OS in the TCGA SKCM cohort (HR = 0.42, 95% CI 0.19-0.94; p = 0.035). Melanoma differentially expressed genes positively associated with high *APRIL/CXCL10/CXCL13* tumor expression were linked to tumor infiltration by a diverse array of proinflammatory immune cell types.

**Conclusion:**

Serum protein and tumor transcript levels of APRIL/TNFSF13 are associated with improved survival outcomes. Patients exhibiting high coordinate expression of *APRIL/CXCL10/CXCL13* transcripts in their tumors displayed superior OS. Further investigation of TLS-kine expression profiles related to clinical outcomes in larger cohort studies is warranted.

## Introduction

Tertiary lymphoid structures (TLS) are lymphoid cell aggregates resembling lymph nodes but found in peripheral tissues impacted by chronic inflammation (1).TLS are frequently observed in the setting of autoimmune disease, and more recently, the identification of TLS in tumors at baseline and within on-treatment tissue samples has been reported to be prognostic of improved overall survival and patient responsiveness to treatment with immune checkpoint blockade (ICB) (2–4). In the setting of resectable melanomas, patients with tumors containing B cells and TLS exhibit superior response to interventional ICB than patients lacking these immune infiltrates (5).

Neogenesis of TLS and lymph nodes are believed to be driven by an overlapping, but not identical set of soluble mediators, i.e., lymphoid chemokines: CCL19, CCL21, and CXCL13, and lymphoid cytokines such as lymphotoxin (LT)-α, LT-αβ, and additional members of the tumor necrosis factor superfamily. Together, these TLS-kines and B cell survival factors including APRIL/TNFSF13 and BAFF/TNFSF13B promote the recruitment and orchestration of T and B lymphocytes, follicular dendritic cells, and vascular elements (i.e., high endothelial venules; HEV) in TLS (2, 6).

The anatomic location of TLS is variable in tissues impacted by cancer, with most of these structures developing in the peritumoral space. This geographic variability and the varying numbers of TLS in melanoma patients with multifocal disease have complicated the reliability of applying tumor tissue imaging techniques as a stand-alone, comprehensive method to identify/monitor these anatomic biomarkers in clinical practice (7, 8). A 12-chemokine gene expression signature including *CCL2-5, CCL8, CCL18, CCL19, CCL21, CXCL9-11*, and *CXCL13* has been previously reported to serve as a predictor for the presence of TLS in melanoma specimens in association with improved OS (7). While the potential clinical utility of tissue profiling of TLS-associated chemokines/cytokines (TLS-kines) transcripts has been described in melanoma patients, serum protein analyses of TLS-kines as informative biomarkers have not been analyzed in depth. In the current study, we performed serum protein and tissue transcriptomic analyses of TLS-kine expression to correlate these findings with clinicopathological/immunological characteristics in a melanoma patients-matched manner, including the presence/absence of tumor-associated lymphoid aggregates (LA) and TLS *in situ*. We observed that melanoma patients with coordinately high serum levels of APRIL and coordinate high APRIL/CXCL10/CXCL13 we're associated with improved survival and proinflammatory TME.

## Materials and methods

### Data source and study population

Patients specimens were obtained from the Melanoma Center of the University of Pittsburgh Medical Center Hillman Cancer Center Biospecimen Repository under the IRB-approved University of Pittsburgh Cancer Institute Melanoma Center Human Biological Sample and Nevus Image Banking and Analysis Protocol (HCC 96-99). Patients with a histological diagnosis of melanoma, known male/female status, age ≥ 18 years old, and availability of serum banked in the biospecimen repository were included in this study. Informed consent was obtained prior to biological specimen collection and banking. Demographic data (age, sex), presence/absence of baseline autoimmune diseases, clinical data (stage, location, date of diagnosis, follow-up time, presence/absence of recurrence/progression/death, lactate dehydrogenase (LDH) levels, and pathological data (histological subtype, degree of tumor-infiltrating lymphocytes, Breslow thickness, presence/absence of ulceration) were collected from electronic medical records and pathological reports. The Cancer Genomic Atlas melanoma cohort (TCGA-SKCM) was used for tissue transcriptomic analyses, with these results then validated using the Moffitt Melanoma cohort. The characteristics of TCGA SKCM and Moffitt Melanoma cohorts have been previously described (9).

### Analysis of serum TLS-kine protein levels and their clinical correlates in melanoma patients

After peripheral blood draw, patient baseline serum was separated from the clot and immediately aliquoted into sterile cryovials in a laminar flow hood and frozen at -80°C. Concentrations of CXCL10, CXCL13, CX3CL1, lymphotoxin-alpha (LTA), CCL19, CCL21, APRIL/TNFSF13, and BAFF/TNFSF13B were simultaneously measured in patients sera using a custom ProcartaPlex panel and Luminex xMAP technology (ThermoFisher Scientific) at the University of Pittsburgh Luminex Core Laboratory.

The primary endpoint of this study was event-free survival measured as time from diagnosis to time of disease recurrence or progression for resectable vs. unresectable/advanced melanoma, respectively. Secondary endpoints were overall survival (time from diagnosis to time of death), and association between TLS-kine levels and clinicopathological characteristics of disease. Chemokine/cytokine levels were log-transformed to make their distribution approach normality. The associations between levels of target analytes and event-free survival (recurrence and/or progression) and overall survival were examined using univariate and multivariate Cox proportional hazards models. The correlations between analytes and clinicopathological variables were examined by Spearman rank correlation coefficient for continuous variables, and Wilcoxon rank-sum and Kruskal-Wallis tests for categorical variables.

### Multispectral immunofluorescence and image analysis

Formalin-fixed paraffin-embedded tissue was sectioned, and mounted onto slides (4 microns). Slides were baked in a dry oven at 60°C, de-paraffinized with xylene and ethanol, and finally re-fixed in 10% neutral buffered formalin for 15 minutes. Whole slides were imaged on Vectra Polaris at 20x slides and were prepared according to manufacturer instructions for MoTIF Vectra panels (Akoya Biosciences). Slides were manually stained in 1 batch, mounted, and delivered to the University of Pittsburgh TPIL core facility for further processing. One section underwent all antigen retrieval procedures but received no antibody treatment and was used as a tissue-specific unstained control for estimation of autofluorescence. Microwave heat-induced antigen retrieval (HIER) was performed, followed by blocking for 10 minutes. Primary antibodies were incubated for 30 minutes at room temperature. Secondary antibodies conjugated to horseradish peroxidase were then added for 10 minutes. Cells were stained with the following primary antibody/conjugated opal pairs: PNAd/Opal690, CD4/Opal570, AID/Opal520, CD21/OpaL480, Ki67/Opal620 and CD20/Opal780 (**Supplementary Table S1**). Nuclei were stained with DAPI and slides were coverslipped and sealed with Pro-Long Diamond Anti-fade mounting media (ThermoFisher). Digital whole slide images were analyzed using Phenochart, and regions with visible TLS-like structures were selected for downstream analysis. ROIs were loaded into Akoya’s InForm Image Analysis software. Autofluorescence was isolated, then a tissue segmentation, cell segmentation, and phenotyping algorithm were developed using a training set of ROIs. This algorithm was subsequently applied in batches to all ROIs selected. Quantification of cell types was performed using the R package Phenotpr. On the H&E assessment lymphoid aggregates (LA) were defined as discrete collections of ≥ 50 lymphocytes, and TLS were defined as LA encompassing HEV and germinal centers. For mIF assessment, we further distinguished TLS containing PNAd^+^ HEV and TLS with a germinal centers containing CD21^+^ FDC networks.

### Gene expression profiling of *APRIL/TNFSF13, CCL19, CXCL10 and CXCL13* associated transcripts in TCGA-SKCM specimens

Patients clinicopathological and mRNA expression data for *APRIL/TNFSF13, CCL19, CXCL10* and *CXCL13* were retrieved from the TCGA-SKCM pan-cancer atlas version (n = 448) using the cBioportal database (https://www.cbioportal.org/). After excluding patients lacking RNA-seq data or survival data, the selected sample included 426 patients which was used to assess the association between GEP profiles for TLS-kines APRIL*/TNFSF13, CCL19*, *CXCL10* and/or *CXCL13* and patients overall survival (OS). The median expression for each gene was used as the cut-off point to classify patients into high and low-expression groups. The association between GEP of *TLS-kines* and OS was also studied in a Moffitt Melanoma cohort (n = 134).

### Functional enrichment analyses

To understand the potential significance of APRIL/TNFSF13 in melanoma, we conducted gene ontology (GO) analyses to assess the enrichment of biological processes (BP), cellular components (CC), and molecular function (MF) of positively and negatively co-expressed genes with *APRIL/TNFSF13*. Kyoto Encyclopedia of Genes and Genomes (KEGG) pathway enrichment analysis was also conducted. In addition, functional enrichment analysis was performed to identify differentially expressed genes (both up- and down-regulated genes) in patients with High vs. Low coordinate expression of CXCL13/TNFSF13/CXCXL10. GO and KEGG enrichment analyses were carried out using the DAVID bioinformatics tool (https://david.ncifcrf.gov/home.jsp). Statistical significance for term enrichment was set at a Benjamini-Hochberg adjusted p value less than 0.05.

### Analysis of patients TME

The infiltration levels of immune cell subsets were identified using the xCell algorithm (https://xcell.ucsf.edu/), the CIBERSORT algorithm (https://cibersortx.stanford.edu/) and the TIDE algorithm was used to quantify myeloid-derived suppressor cells (MDSC) (http://tide.dfci.harvard.edu/). TLS-kine correlations with cell infiltration levels were assessed using Spearman’s Rho correlation coefficient. The expression pattern of TLS-kine genes in tumor and immune cells was further investigated using single-cell RNA analysis through the Curated Cancer Cell Atlas (https://www.weizmann.ac.il/sites/3CA/) (10). We analyzed two single-cell datasets from the Gene Expression Omnibus (GEO) involving melanoma patients under the accession numbers GSE115978 and GSE72056 using the Seurat package.

## Results

### Melanoma patient baseline characteristics and serum TLS-kine protein profiling

This study involved serum analyses of 95 melanoma patients. Forty-eight patients (50%) were females, the median age for all patients evaluated was 63, and the interquartile range (IQR) for the entire cohort was 51-70 years (**Table 1**).

**Table 1:** Melanoma patient demographics and clinical characteristics.

Variable	Median (IQR) or number (%)
Age (years)	63 (51-70)
Gender	
Male	47 (50%)
Female	48 (50%)
Autoimmune disease	
Yes	20 (21%)
No	75 (79%)
Stage	
Stage I	26 (27%)
Stage II	10 (11%)
Stage III	15 (16%)
Stage IV	44 (46%)
LDH	188 (170-237)
Location	
Trunk	30 (32%)
HN	17 (18%)
LE	16 (17%)
UE	17 (18%)
Other	14 (15%)
Primary treatment	
Surgery	43 (45%)
Systemic therapy	52 (55%)
Serum APRIL/TNFSF13 (pg/ml)	584 (395-803)
Serum CCL19 (pg/ml)	156 (97-220)
Serum CXCL10 (pg/ml)	9 (6-12)
Serum CXCL13 (pg/ml)	78 (56-142)

LDH, Lactate dehydrogenase; HN, Hilar nodes; LE, Lower extremity; UE, Upper extremity; IQR, Interquartile-range.

Most patients had stage IV disease (44 of 95; 46%), followed by stage I disease (26 of 95; 27%), stage III disease (15 of 95; 16%), and stage II disease (10 of 95; 11%). Most melanomas (38 of 95; 40%) had a superficial spreading histology, with 23 patients (24%) presenting with nodular histology. Thirty melanomas (32%) were located on the trunk, with 16 (17%) isolated from lower extremities. Forty-three (45%) patients developed disease recurrence or progression, with 27 (28%) patients having died without event with a median follow-up time of 56 months. Overall, 89%, 92%, 82%, and 50% of patients failed to display detectable serum levels of BAFF/TNFSF13B, CX3CL1, LTα, and CCL21 (defined as < 2 pg/ml, < 0.5 pg/ml, < 1 pg/ml and <7 pg/ml for the respective analytes) amongst an initial cohort of 78 melanoma patients evaluated. The remaining analytes were identified at detectable levels in all patients analyzed (N = 95), including APRIL/TNFSF13 [median IQR = 584 pg/ml (range 395-803)], CXCL10 [9 pg/ml (6-12)], CXCL13 [78 pg/ml (56-142)], and CCL19 [156 pg/ml (97-220)]. These latter four TLS-kines which were detected in patients’ serum over a dynamic concentration range became the focus of extended analyses.

### Correlations between serum TLS-kine protein levels and patients demographic and clinicopathological characteristics

**Figure 1:**
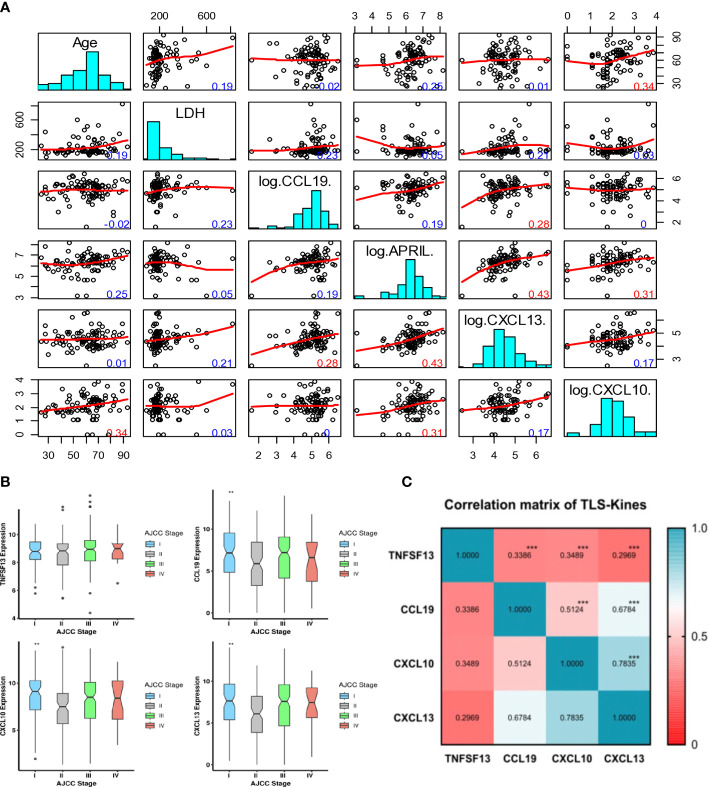
Intervariable correlation between serum and tissue transcripts of TLS-kines and patients clinical indices. **(A)** Scatterplot matrix showing correlation between individual TLS-kines levels, LDH serum levels or patients Age. The y-axis in each plot representing age (row 1), LDH levels (row 2), CCL9 (row 3). APRIL (row 4), CXCL13 (row 5), CXCL10 (row 6) and the x-axis being the intersecting factor from adjacent rows. Red numbers highlight significant correlations (p < 0.01), blue numbers are not significant. **(B)** Box plots illustrating TLS-kines expression based on patients’ AJCC stage. CCL19, CXCL10 and CXCL13 transcript expression levels were significantly associated with tumor stage in the TCGA-SKCM cohort. P values were determined using the analysis of variance test (ANOVA). All p values < 0.05 were considered as significant. **(C)** Correlation of individual TLS-kine transcript levels with each other. Correlation matrix showing the association between TLS-kines in the TCGA-SKCM cohort. * p <.05, ** p <.01, *** p <.001. Each of these TLS-kines was significantly positively correlated with each other, with CXCL10 and CXCL13 having the highest correlation coefficient for coexpression (Pearson’s r = 0.783, p < 0.001).

Patients’ age was positively correlated with serum levels of CXCL10 (r = 0.34, p ≤ 0.01) but not with other TLS-kines evaluated (**Figure 1A**). There was a trend towards higher serum levels of CXCL13 amongst male patients (p = 0.0525), with no significant gender-based differences observed for the other analytes. The presence of known baseline autoimmune disease (psoriasis, hypothyroidism, ulcerative colitis, rheumatoid arthritis, celiac disease, Graves’ disease, systemic lupus erythematosus) was not associated with (high) patients’ serum levels of any TLS-kine evaluated in our study (all p > 0.05).

Serum levels of APRIL/TNFSF13, CXCL10 and CXCL13 did not vary by disease stage (p > 0.05). However, patients’ serum levels of CCL19 were observed to vary as a function of disease stage (p = 0.02), with the highest levels detected in stage III and IV patients. Tumors with brisk/non-brisk tumor-infiltrating lymphocytes (TIL) vs. absence of TIL exhibited significantly higher levels of APRIL/TNFSF13 (p = 0.01), CCL19 (p = 0.01) and CXCL13 (p = 0.01), but no such difference was observed for patients’ serum levels of CXCL10 (p = 0.97) (**Figure S1**). There was no significant difference in patients’ serum levels of APRIL, CCL19, CXCL10, or CXCL13 as a function of melanoma histological subtype. Levels of serum TLS-kines were not correlated with patients’ levels of LDH, a marker of systemic tissue pathology. Remarkably, serum levels of APRIL/TNFSF13 were positively correlated with levels of both CXCL10 and CXCL13 (r = 0.31 and r = 0.43, respectively; p ≤ 0.01) (**Figure 1A**). There was also a weaker but significant correlation between patients’ serum levels of CCL19 and CXCL13 (r = 0.28, p ≤ 0.01). Only serum *CXCL10* levels exhibited significant differences across patients harboring melanomas with different mutations (BRAF, CDKN2A, NF, NRAS) (**Figure S2A**).

### Serum APRIL/TNFSF13 levels correlate with improved event-free survival

In a univariate analysis, increasing patients age (p = 0.019), disease stage (p = 0.0001) and LDH levels (p = 0.0027) were negatively associated with patients’ event-free survival. Conversely, levels of serum APRIL/TNFSF13 were positively associated with improved patients’ event-free survival (**Table 2**).

**Table 2:** Univariate proportional hazards regression for event - free survival.

Covariate	HR	95% CI	p-value
Age	1.63	1.08 – 2.45	0.0188
Gender Male	1.47	0.80 – 2.69	0.2109
Stage	1.3	0.26 – 6.45	0.0001
II	3.7	1.03 – 13.26	
III	9.49	3.18 – 28.36	
IV			
LDH	1.32	1.10 – 1.60	0.0027
Treatment Surgery	0.14	0.06 - .33	<0.0001
Location	1.05	0.46 – 2.40	0.1934
HN	0.45	0.16 – 1.24	
LE	0.49	0.21 – 1.12	
UE	l.28	0.42 – 3.84	
Other			
Serum APRIL/TNFSF13^Hi^	0.64	0.43 – 0.95	0.0285
Serum CCL19^Hi^	1.2	0.88 – 1.64	0.2453
Serum CXCL10^Hi^	1.213	0.91 – 1.42	0.2616
Serum CXCL13^Hi^	0.81	0.59 – 1.10	0.1785

LDH, Lactate dehydrogenase; HN, Hilar nodes; LE, Lower extremity; UE, Upper extremity.

In a multivariate analysis after adjusting for age and stage, serum APRIL/TNFSF13 levels remained significantly associated with improved patients event-free survival (HR = 0.48, 95% CI 0.31-0.78; p = 0.025). In a multivariate analysis, age and disease stage were associated with worse overall survival, and elevated serum APRIL/TNFSF13 levels trended as being associated with improved overall survival (p = 0.056). LDH was initially included in the multivariate model but was found to be uninformative above age, stage and APRIL expression (HR: 1.03, p = 0.7507). There were no significant associations identified between serum levels of the individual chemokines CCL19, CXCL10 or CXCL13 and patients’ event-free or overall survival.

### Coordinate expression of *APRIL/TNFSF13, CXCL10* and *CXCL13* transcripts is associated with superior patient overall survival in the TCGA-SKCM cohort

**Figure 2:**
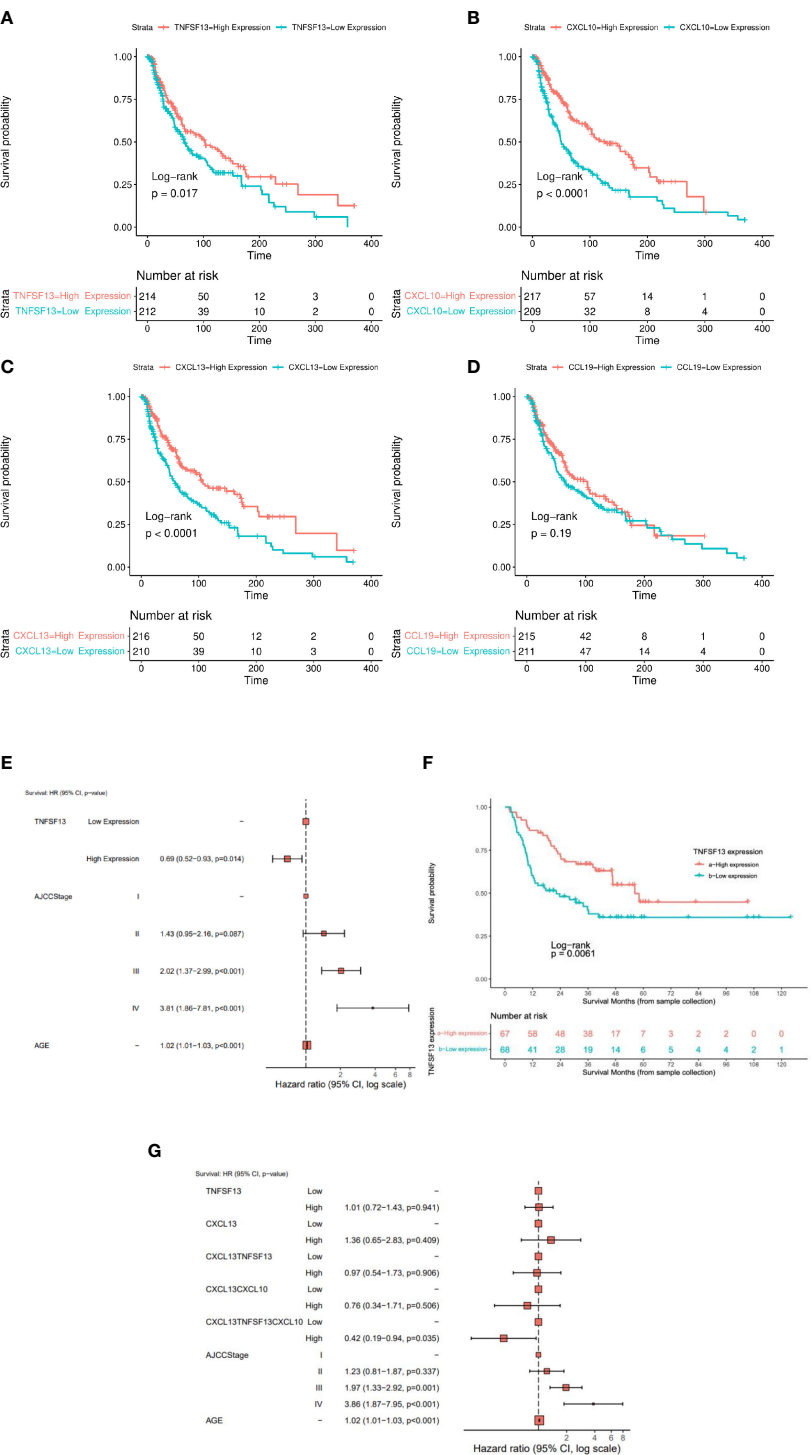
The expression of TLS-kines in association with patients clinical outcome. Kaplan Meier plots depicting OS probability for TCGA-SKCM (n = 426) patients stratified based on expression of **(A)** *APRIL/TNFSF13*, **(B)** *CXCL10*, **(C)** *CXCL13* or **(D)** *CCL19*. High expression of *APRIL/TNFSF13* (HR: 0.72 95%, CI: 0.55-0.94; p = 0.018), *CXCL13* (HR: 0.58 95%, CI: 0.44-0.76; p < 0.001), *CXCL10* (HR: 0.51, 95% CI: 0.39-0.68; p < 0.001) were significantly associated with improved OS in TCGA-SKCM patients. **(E)** Cox multivariable model after adjusting for age, AJCC stage and *APRIL/TNFSF13* expression. **(F)** Kaplan Meier plot of *APRIL/TNFSF13* expression in Moffit cohort. High expression showed a significant better OS than lower expression (p-value < 0.05). **(G)** Forest plot for the Cox proportional hazard model when adjusting for *CXCL13, APRIL/TNFSF13*, the average expression groups of *CXCL13* + *APRIL/TNFSF13*, the average expression groups of *CXCL13 + APRIL/TNFSF13* + *CXCL10*, AJCC Stage and age. The average expression of the combined gene signature *APRIL/TNFSF13* + *CXCL10* + *CXCL13* showed an independent prediction of OS along with age and AJCC stage (p-value < 0.05).

*CCL19, CXCL10*, and *CXCL13* transcript expression levels were significantly associated with tumor stage (lowest levels in stage 2) in the TCGA-SKCM cohort (**Figure 1B**). Each of these TLS-kines were significantly positively correlated with each other, with *CXCL10* and *CXCL13* having the highest correlation coefficient in paired comparisons (Pearson’s r = 0.783, p < 0.001) (**Figure 1C**). *BRAF* mutated patients had significantly higher expression of *APRIL*/*TNFSF13* and *CXCL10* in comparison to *BRAF* wild-type patients, while *NRAS* wild-type patients had higher expression of *TNFSF13* in comparison to patients with melanomas harboring *NRAS* mutations (**Figure S2B**). High expression of *APRIL/TNFSF13* (HR = 0.72, 95% CI: 0.55-0.94, p = 0.017), *CXCL10* (HR = 0.51, 95% CI: 0.39-0.68; p < 0.0001) and *CXCL13* (HR = 0.58, 95% CI: 0.44-0.76; p < 0.0001) were significantly associated with improved OS in TCGA-SKCM patients (**Figures 2A–C**). Whereas CCL19 expression did not show any significant association with survival (HR = 0.83, 95% CI: 0.63-1.09, p = 0.187) (**Figure 2D**). In multivariable analyses after adjusting for age and stage, *APRIL/TNFSF13* remained significantly associated with OS (HR = 0.69, 95% CI 0.52-0.93; p = 0.014) (**Figure 2E**). High expression of *APRIL/TNFSF13* was also significantly associated with improved OS in Moffitt melanoma patients (APRIL/*TNFSF13*: HR = 0.51, 95% CI: 0.32-0.82; p = 0.006) (**Figure 2F**). A multivariable overall survival model was then constructed to assess the prognostic performance of the coordinate expression of *CXCL13* and other TLS-kines with significant OS associations in univariable analyses (*APRIL/TNFSF13, CXCL10*) as well as age and stage adjustment. CXCL13 was used as the core for each of the combinations based on its established role in (mature) TLS formation. We determined that the 3-gene index of *APRIL/TNFSF13*, *CXCL10* and *CXCL13* was the only significant classification after multivariable adjustment (HR = 0.42, CI: 0.19-0.94; p = 0.035) (**Figure 2G**; **Supplementary Table S2**). These data suggest that amongst the TLS-kines evaluated, coordinate high expression of *APRIL/TNFSF13, CXCL10* and *CXCL13* transcripts may represent a minimal TLS-kine index predictive of superior OS in melanoma patients.

### *APRIL/TNFSF13-* and combined *TNFSF13/CXCL10/CXCL13*-associated functional gene enrichment analyses

**Figure 3:**
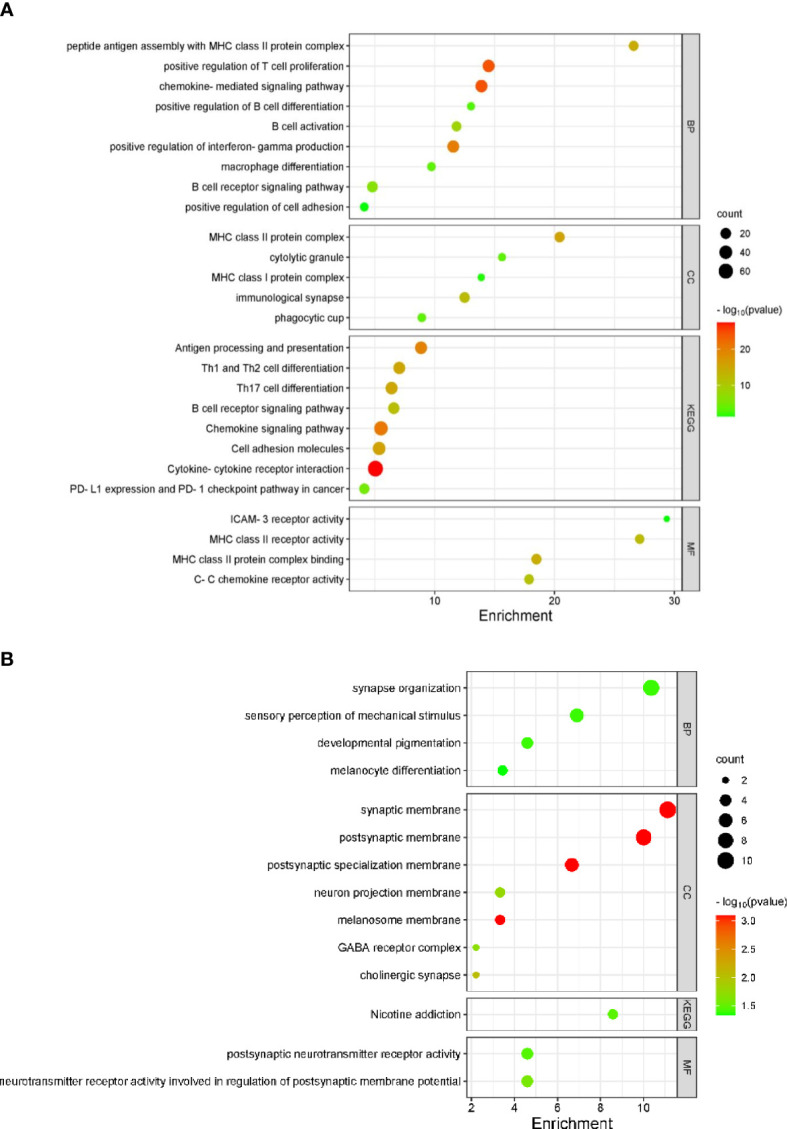
TNFSF13/CXCL10/CXCL13 associated gene pathways in TCGA melanoma specimens (n = 426). Bubble plots identifying significantly enriched GO terms for BP, CC, MF and KEGG pathways for upregulated **(A)** and downregulated **(B)** genes in PTs with high TNFSF13/CXCL10/CXCL13 transcript expression.

Genes coordinately expressed with *APRIL/TNFSF13* were identified through cBioportal (**Supplementary Table S3**). The top 10 genes positively correlated with *APRIL/TNFSF13* expression in the TME included *CD4, ITGAM, SLC7A7, LAIR1, ITGB2, SELPLG, TMEM176B, HCK, TMEM176A*, and *CYTH4*, while the top 10 negatively correlated genes were *COA7, WDR43, NCL, PAICS, WDR12, NOLC1, TEX10, EXOSC2, MSANTD3 and CCDC43*. GO analyses for the top 100 positively correlated genes linked to *APRIL/TNFSF13* revealed enrichment for several terms including the BP terms ‘positive regulation of T cell activation’ and ‘antigen processing and presentation of exogenous peptide antigen *via* MHC class II’, the CC terms ‘MHC class II protein complex’ and ‘immunological synapse’ and the MF terms ‘MHC class II protein complex binding’ and ‘signaling receptor activity’, while KEGG pathway analyses identified ‘Antigen processing and presentation’, ‘Cell adhesion molecules’ pathways to be significantly enriched (**Supplementary Table S4**; **Figure S3A**). Among the top 100 negatively correlated genes associated with *APRIL/TNFSF13 transcript expression*, the BP term ‘rRNA processing’, the CC terms ‘mitochondrion’, the MF term ‘RNA binding’, as well as the KEGG pathway ‘Ribosome biogenesis in eukaryotes’ were significantly enriched (**Supplementary** **Table S4**; **Figure S3B**). Following gene differential expression analysis for patients with *TNFSF13/CXCL10/CXCL13* high and low expression, the top 10 upregulated and downregulated genes are shown in **Supplementary Table S5**. GO analyses for the combined *TNFSF13/CXCL10/CXCL13* high patients cohort revealed enrichment for several terms in upregulated genes including the BP terms ‘B cell activation’, ‘positive regulation of T cell proliferation’, and ‘positive regulation of interferon-gamma production’, the CC terms ‘MHC class II protein complex’ and ‘immunological synapse’ and the MF terms ‘C-C chemokine receptor activity’ and ‘MHC class II receptor activity’, and KEGG pathway ‘cytokine-cytokine receptor interaction’, ‘Th1, Th2, and Th17 cell differentiation’ (**Figure 3A**). Among downregulated genes associated with high *TNFSF13/CXCL10/CXCL13* co-expression, the BP term ‘sensory perception of mechanical stimulus’, the CC terms ‘cholinergic synapse’, ‘neuron projection membrane’, ‘GABA receptor complex’ as well as the MF term ‘postsynaptic neurotransmitter receptor activity’ were enriched (**Figure 3B**).

### High expression of *APRIL/CXCL10/CXCL13* in tumors is linked to elevated proinflammatory immune cell infiltration

**Figure 4:**
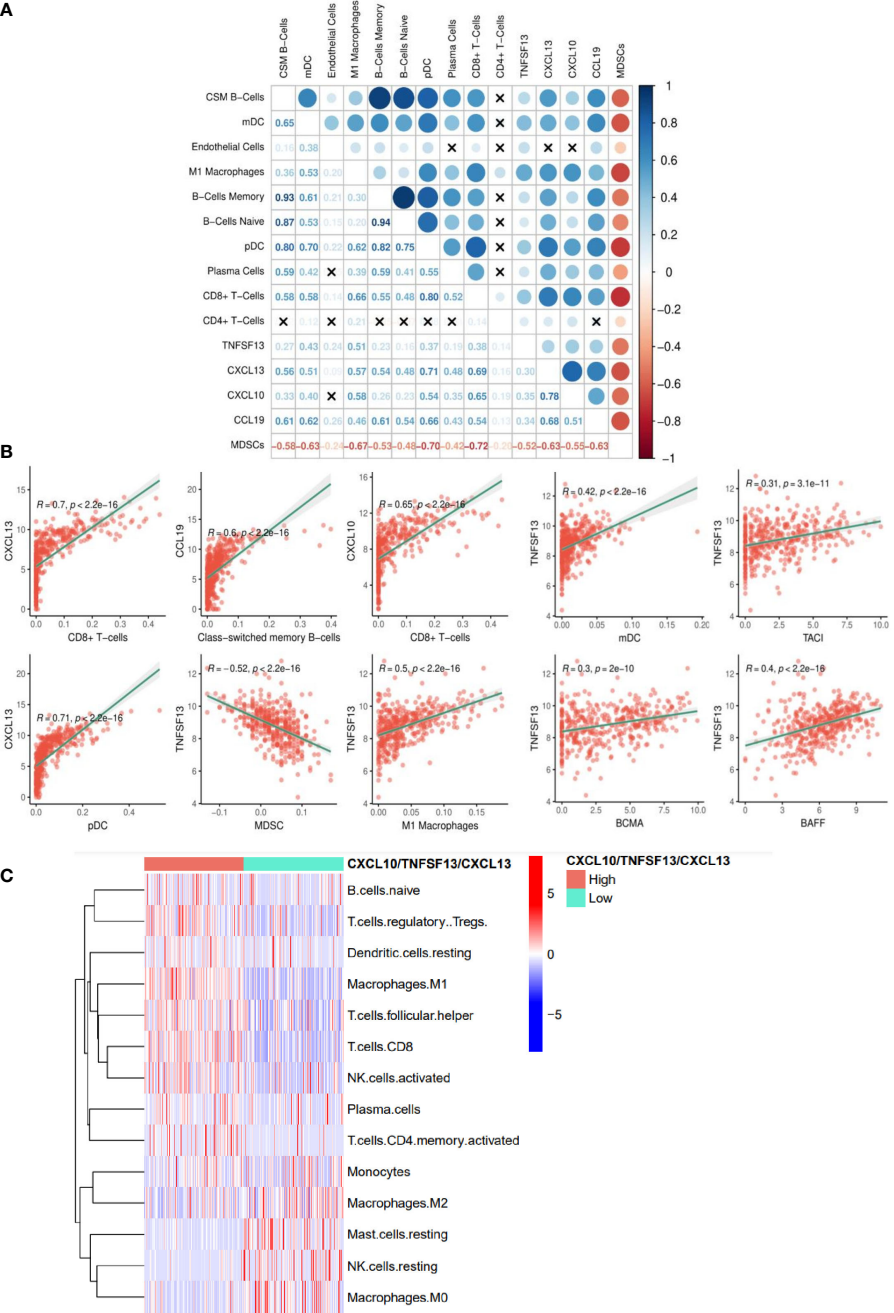
TLS-kine gene expression correlates in the melanoma TME. **(A)** TLS-kine correlation with the infiltration levels of adaptive immune cells in patient melanoma specimens. **(B)** Scatter plot demonstrating the association between TLS-kine gene transcripts and the infiltration level of immune cells of interest assessed using the xCell algorithm and the TIDE algorithm for MDSCs. Correlation r and p values are depicted on the figure. *CXCL13* expression was positively correlated with CD8^+^ T cell and plasmacytoid dendritic cell (pDCs) content in the TME. *CXCL10* expression was positively correlated with CD8^+^ T cell content in melanomas. *CCL19* expression was positively correlated with tumor-infiltrating levels of class-switched memory B cells. *APRIL/TNFSF13* expression was significantly positively correlated with tumor content of M1 macrophages, myeloid dendritic cells (mDCs), and expression of *BAFF, TACI and BCMA*, but negatively correlated with MDSC content. **(C)** A heat map reveals immune cell infiltration type using the CIBERSORT deconvolution algorithm with statistically significant differences amongst high patients exhibiting high vs. low coordinate expression of *APRIL/TNFSF13* + *CXCL10* + *CXCL13*.

Each of the individual TLS-kines were significantly positively correlated with levels of melanoma infiltration by CD8^+^ T cells, with *CXCL13* having the strongest association (r = 0.7) (**Figure 4A**). *APRIL/TNFSF13* expression was significantly positively correlated with tumor content of M1 macrophages (r = 0.5, p < 0.0001), myeloid DCs (r = 0.42, p < 0.0001) and negatively correlated with MDSC (r = -0.52, p < 0.0001) content. Class-switching memory B cells and DCs (both plasmacytoid and myeloid) were also strongly correlated with levels of TLS-kine transcripts in the TME (**Figure 4B**). The expression of *APRIL/TNFSF13* was positively correlated with that of its receptors *TACI/TNFSFR13B* (r = 0.31, p < 0.0001) and *BCMA/TNFSFR17* (r = 0.30, p < 0.0001) and with *BAFF/TNFSF13B* (r = 0.40, p < 0.0001) in the TCGA cohort (**Figure 4B**), suggestive of the operational relevance of these biologic circuits *in situ*. Melanoma patients with coordinately high expression of the *TNFSF13, CXCL10*, and CXCL13 exhibited significantly higher infiltration by core TLS cell populations including naïve B cells, plasma cells, CD8^+^ T cells and M1 macrophages, while melanomas deficient in *TNFSF13/CXCL10/CXCL13* transcripts were enriched in M2 macrophages and mast cells (**Figure 4C**). Single-cell RNh21A sequence data supported *in situ* expression of *APRIL/TNFSF13* predominantly by tumor-associated macrophages (**Figures S4A, B**), a finding which was consistently observed across a range of alternate solid tumor types (**Figure S4C**). *CXCL10* was also expressed predominantly by macrophages in the TME, while *CXCL13* was mainly expressed by CD8^+^ TIL (**Figures S4D, E**).

In an exploratory fashion, we further studied melanomas from 5 patients with coordinately elevated serum levels of TNFSF13 and CXCL10 or CXCL13. Tumors from each of these patients contained LA and/or TLS, with a distribution of 27.1% TLS, 4.17% TLS with germinal centers, and 68.75% LA observed in a total of 48 melanoma tissue sections evaluated (**Supplementary Table S6**; **Figure S5**). While all 5 patients also demonstrated > lower limit of quantification (LLOQ) levels of CCL19, only two patients had detectable levels of CCL21, only one patient had a >LLOQ level of BAFF/TNFSF13B, and none of these patients had detectable serum levels of CX3CL1. Hence, this small pilot study supports a paradigm for studying expression of a 3 TLS-kine index (APRIL/TNFSF13, CXCL10 and CXCL13) in serum that may predictive presence of histologically defined LA and/or TLS in the TME of melanoma patients.

Together these data suggest that 3-component index that integrates high expression of APRIL/TNFSF13, CXCL10, and CXCL13 transcripts in tumor are associated with superior melanoma patients' survival and a pro-inflammatory TME supportive of LA and /or TLS.

## Discussion

This study investigated clinical correlates of TLS-associated cytokines/chemokine expression levels in serum (protein) and tumor (transcript) specimens isolated from patients with melanoma. Our work revealed that serum level of APRIL/TNFSF13, but not CCL19, CXCL10 or CXCL13 when considered as single analytes, was associated with improved event-free survival (EFS) in patients with melanoma. Serum level of APRIL/TNFSF13 remained significantly associated with EFS and trended to be associated with OS after adjusting for age and stage. High *APRIL/TNFSF13* transcript expression was also significantly associated with improved OS in both the TCGA SKCM and Moffitt Melanoma patient cohorts.

A proliferation-inducing ligand (APRIL/TNFSF13/CD256) is a member of the TNFSF superfamily of molecules. APRIL/TNFSF13 and BAFF/TNFSF13B bind to the TACI/TNFSFR13B and BCMA/TNFSFR17 receptors, and play major roles in the recruitment, maturation, differentiation and survival of B cells, as well as the anti-tumor efficacy of DCs (11–13). APRIL/TNFSF13 is expressed by macrophages, lymphoid cells, as well as tumor cells and keratinocytes, and interestingly, expression of *APRIL/TNFSF13* is commonly downregulated in progressive melanomas when compared to normal skin tissue (13, 14). In support of our results, *APRIL/TNFSF13* was recently identified as one of 8 genes associated with improved prognosis of patients with melanoma based on analyses of TCGA and GEO databases (15). Although not detectable in the serum of most patients in our study, BAFF/TNFSF13B has been reported to suppress melanoma growth by potentiating Type-1 monocytes, activated T cells and local production of proinflammatory cytokines while mitigating the suppression mediated by regulatory cell populations, leading to enhanced anti-tumor immunity in melanoma models (16, 17).

Differentially expressed genes (DEGs) in melanomas that were positively associated with *APRIL/TNFSF13* expression included the immune cell biomarkers (*CD4, CD11b/ITGAM, LAIR1, LFA-1/ITGB2*) and several genes linked to immune cell infiltration of tumors, including *CYTH4, SLC7A7/LAT1, SELPLG, TMEM176A*, and *TMEM176B*. The CD11b/ITGAM integrin is expressed by macrophages, granulocytes, and subsets of NK cells, B cells, and CD8^+^ T cells and its expression is prognostic of improved patient response to ICB in lung cancer patients (18–20). *LAIR1* expression has been reported to be prognostic of improved clinical outcomes in patients with advanced melanoma and with macrophage content in the TME (21, 22). LFA-1/ITGB2 is an important immune cell integrin that is a biomarker of tumor inflammatory infiltrates (20). In the setting of ovarian carcinoma, *CYTH4* expression is linked to tumor-infiltrating immune cell content and local expression of proinflammatory chemokines and antigen-presentation machinery in the TME (23). *SLC7A7/LAT1* is a biomarker of superior immune infiltration (B cells, CD4^+^ and CD8^+^ T cells, DCs, macrophages, and neutrophils) and improved clinical outcomes in patients with non-small cell lung cancer (NSCLC) (24). Expression of SELPLG (P selectin ligand) in the TME is correlated with increased melanoma infiltration by B cells, CD4^+^ and CD8^+^ T cells, DCs, and macrophages (25). *TMEM17A* and *TMEM176B* are tumor suppressor genes whose expression is positively correlated with increased T cell infiltration and better overall survival in patients with melanoma or gastric cancer (26–28). Melanoma DEGs negatively associated with *APRIL/TNFSF13* expression included known oncogenes correlated to poor clinical outcomes such as *NCL/nucleolin, NOLC, WDR12*, and *WDR43* (29–32). *CCDC43* expression is predictive of poor prognosis and metastatic spread in patients with colorectal cancer, gastric cancer or oral squamous cell carcinoma (OSCC) (33–35). In patients with OSCC, high expression of *CCDC43* correlates with reduced tumor infiltration by B cells, DCs, CD8^+^ T cells and natural killer T cells (35). *EXOSC2* has been reported to represent a biomarker of poor clinical prognosis and reduced response to interventional therapy with ICB in NSCLC patients (36). *NOLC1, PAICS*, and *TEX10* expression are associated with cancer stemness, poor prognosis, and resistance to interventional chemo/radiotherapy in patients with breast cancer, esophageal cancer, hepatocellular carcinoma or melanoma (25, 37–41). These data support an *APRIL/TNFSF13*-enriched TME as proinflammatory and characteristic of extended EFS/OS, while an *APRIL/TNFSF13*-deficient TME excludes immune cells in support of disease progression and poor clinical outcome. These findings were confirmed for serum levels of APRIL/TNFSF13 which were increased in patients with tumors containing brisk or non-brisk TIL vs. absent, but were not correlated with disease stage, histological subtypes of melanoma, or circulating levels of LDH. The patients with high coordinate expression of *TNFSF13/CXCL10/CXCL13* as opposed to *CXCL13* alone (which has previously served as a surrogate biomarker for TLS formation) (4, 42) exhibited superior OS. Notably, high coordinate expression of the *TNFSF13/CXCL10/CXCL13* transcripts in melanomas was correlated with an increased presence of naïve B cells, plasma B cells, CD8^+^ T cells, and M1 macrophages and decreased levels of M2 macrophages and mast cells as well as a reduced neural network gene signature. This latter aspect is intriguing given recent publications reporting the immunoregulatory nature of sensory neurons which may operationally prevent TLS formation in the melanoma TME *in vivo* (43, 44).

Overall, these data support further investigation of coordinate APRIL/TNFSF13, CXCL10 and CXCL13 expression at the serum protein and tumor transcript levels as indices for monitoring LA/TLS status in patients’ melanomas at baseline vs. on-treatment with interventional (immune)therapies as predictors of favorable clinical outcomes. Further prospective validation of these findings to reach definitive conclusions in larger cohort studies is warranted.

## Data availability statement

The original contributions presented in the study were obtained from TCGA-SKCM cohort and Moffitt melanoma cohort, further inquiries can be directed to the corresponding authors.

## Ethics statement

The studies involving human participants were reviewed and approved by University of Pittsburgh IRB, Moffitt Cancer Center IRB. Written informed consent for participation was obtained from patients under 96-099 protocol.

## Author contributions

LK, JK, WS: conceptualization, designed experiments, data interpretation, overseeing the overall project, initial writing of manuscript, final writing and editing the manuscript. HA, AA, RD: data analysis (TCGA SCKM). AC, ZT: data analysis (Moffitt Melanoma cohort). WG: data analysis (Pittsburgh cohort). CS, SK, IM, AKn, XY: collection of data, data review, performance of experiments. AL, AKa, DDe: pathology slide review. YN, DDa, JL, JK: provision of study materials. JL, TB: provision of scientific insight. All authors performed final writing, review and editing of the manuscript.
