# Supplementary material for: Expression of lymphoid structure-associated cytokine/chemokine gene transcripts in tumor and protein in serum are prognostic of melanoma patient outcomes
Source: Front Immunol. 2023 Jun 22;14:1171978. doi: 10.3389/fimmu.2023.1171978 (PMC10332263; doi:10.3389/fimmu.2023.1171978)
Supplement: Supplementary file 1 [file DataSheet_1.docx]

**Supplementary Table S1.** Antibodies used for IFM evaluation of lymphoid aggregates and TLS in melanoma tissues.

|  | Manufacturer | product# | Target | clone | AR |
| --- | --- | --- | --- | --- | --- |
| P7HuP82 TLS Maturity | Sigma | MABF2050 | Peripheral Node Addressin (PNAd) | MECA-79 | 9 |
|  | Biocare | API3209AA | CD4 | EP204 | 9 |
|  | Abcam | ab269454 | AID (Anti-AICDA) | EPR23436-45 | 9 |
|  | Leica | NCL-L-CD21-2G9 | CD21 | 2G9 | 6 |
|  | Epredia (Fisher) | RM9106S | Ki67 | SP6 | 6 |
|  | Leica | NC-L-CD20-L26 | CD20 | L26 | 6 |

AR-antigen retrieval; AR9 is high pH buffer; AR6 is low pH buffer

**Supplementary Table S2**. Multivariable overall survival cox regression model for gene combinations with CXCL13 as the core with adjustment for AJCC stage and age.

| Covariate | Group | Mean/N | HR (univariable) | HR (multivariable) |
| --- | --- | --- | --- | --- |
| APRIL/TNFSF13 | Low | 202 (51.7) | - | - |
|  | High | 189 (48.3) | 0.74 (0.56-0.99, p=0.041) | 1.01 (0.72-1.43, p=0.941) |
| CXCL13 | Low | 196 (50.1) | - | - |
|  | High | 195 (49.9) | 0.51 (0.38-0.68, p<0.001) | 1.36 (0.65-2.83, p=0.409) |
| CXCL13 + TNFSF13 | Low | 196 (50.1) | - | - |
|  | High | 195 (49.9) | 0.50 (0.37-0.67, p<0.001) | 0.97 (0.54-1.73, p=0.906) |
| CXCL13 + CXCL10 | Low | 199 (50.9) | - | - |
|  | High | 192 (49.1) | 0.45 (0.34-0.61, p<0.001) | 0.76 (0.34-1.71, p=0.506) |
| CXCL13 + TNFSF13 + CXCL10 | Low | 200 (51.2) | - | - |
|  | High | 191 (48.8) | 0.43 (0.32-0.58, p<0.001) | 0.42 (0.19-0.94, p=0.035) |
| AJCC Stage | I | 85 (21.7) | - | - |
|  | II | 119 (30.4) | 1.72 (1.16-2.56, p=0.007) | 1.23 (0.81-1.87, p=0.337) |
|  | III | 165 (42.2) | 2.10 (1.44-3.08, p<0.001) | 1.97 (1.33-2.92, p=0.001) |
|  | IV | 22 (5.6) | 3.47 (1.72-6.99, p<0.001) | 3.86 (1.87-7.95, p<0.001) |
| AGE | Mean (SD) | 57.8 (15.8) | 1.02 (1.01-1.03, p<0.001) | 1.02 (1.01-1.03, p<0.001) |

 Abbreviations: HR: Hazard ratio

**Supplementary Table S3.** Top 10 positively and negatively correlated genes with tumor expression of APRIL/TNFSF13.

| Positively  correlated genes | Cytoband | Spearman's Correlation | q-Value | Negatively correlated  genes | Cytoband | Spearman's Correlation | q-Value |
| --- | --- | --- | --- | --- | --- | --- | --- |
| CD4 | 12p13.31 | 0.61 | 5E-34 | COA7 | 1p32.3 | -0.45 | 5E-18 |
| ITGAM | 16p11.2 | 0.61 | 5E-34 | WDR43 | 2p23.2 | -0.44 | 3E-17 |
| SLC7A7 | 14q11.2 | 0.60 | 1E-33 | NCL | 2q37.1 | -0.44 | 4E-17 |
| LAIR1 | 19q13.42 | 0.60 | 2E-33 | PAICS | 4q12 | -0.44 | 5E-17 |
| ITGB2 | 21q22.3 | 0.60 | 6E-33 | WDR12 | 2q33.2 | -0.43 | 4E-16 |
| SELPLG | 12q24.11 | 0.60 | 6E-33 | NOLC1 | 10q24.32 | -0.42 | 2E-15 |
| TMEM176B | 7q36.1 | 0.59 | 2E-32 | TEX10 | 9q31.1 | -0.42 | 3E-15 |
| HCK | 20q11.21 | 0.59 | 2E-32 | EXOSC2 | 9q34.12 | -0.41 | 5E-15 |
| TMEM176A | 7q36.1 | 0.59 | 6E-32 | MSANTD3 | 9q31.1 | -0.41 | 6E-15 |
| CYTH4 | 22q13.1 | 0.58 | 2E-31 | CCDC43 | 17q21.31 | -0.41 | 9E-15 |

**Supplementary Table S4**. Significantly enriched GO and KEGG terms for APRIL/TNFSF13 positively and negatively co-expressed genes.

| Upregulated  Class | Term | Enrichment | Adjusted P value | Genes |
| --- | --- | --- | --- | --- |
| BP | inflammatory response | 6.76 | 1.07E-04 | *CSF1R, NFAM1, ITGB2, PTAFR, LY86, CYBB, LYZ, TNFRSF1B, AIF1, HCK, C3AR1, CD14, HAVCR2, CMKLR1* |
|  | positive regulation of T cell activation | 36.09 | 2.06E-04 | *CCDC88B, HLA-DMA, CD4, HLA-DMB, HLA-DPB1, HLA-DRA* |
|  | antigen processing and presentation of exogenous peptide antigen via MHC class II | 33.09 | 0.0020 | *CD74, HLA-DMA, HLA-DMB, HLA-DPB1, HLA-DRA* |
|  | peptide antigen assembly with MHC class II protein complex | 49.63 | 0.0054 | *HLA-DMA, HLA-DMB, HLA-DPB1, HLA-DRA* |
|  | defense response to tumor cell | 45.81 | 0.0498 | *SPI1, ABI3, LAPTM5* |
| CC | MHC class II protein complex | 39.37 | 1.71E-04 | *CD74, HLA-DMA, HLA-DMB, HLA-DPB1, HLA-DRA* |
|  | immunological synapse | 18.20 | 0.0213 | *CD53, IL4I1, HLA-DRA, HAVCR2* |
|  | NADPH oxidase complex | 36.13 | 0.0396 | *NCF2, NCF4, CYBB* |
| MF | MHC class II protein complex binding | 43.10 | 5.02E-05 | *CD74, HLA-DMA, CD4, HLA-DMB, HLA-DPB1, HLA-DRA* |
|  | signaling receptor activity | 6.72 | 0.0198 | *P2RY6, CD4, CSF3R, IL10RA, ITGB2, SLAMF8, SIGLEC7, CMKLR1* |
| KEGG | Cell adhesion molecules | 8.06 | 2.54E-04 | *HLA-DMA, CD4, SELPLG, ITGAM, HLA-DMB, ITGB2, HLA-DPB1, HLA-DRA, VSIR* |
|  | Antigen processing and presentation | 10.82 | 0.0028 | *CD74, HLA-DMA, CD4, HLA-DMB, HLA-DPB1, HLA-DRA* |
|  | Th1 and Th2 cell differentiation | 9.17 | 0.0047 | *HLA-DMA, CD4, HLA-DMB, HLA-DPB1, HLA-DRA, MAPK13* |
|  | Th17 cell differentiation | 7.81 | 0.0085 | *HLA-DMA, CD4, HLA-DMB, HLA-DPB1, HLA-DRA, MAPK13* |
| Downregulated  Class | Term | Enrichment | Benjamini P value | Genes |
| BP | rRNA processing | -11.06 | 0.0235 | *PDCD11, DKC1, HEATR1, DDX21, NOLC1, EXOSC2, WDR43* |
| CC | nucleolus | -3.75 | 4.03E-06 | *UTP25, PANK1, WDR3, SNORD1C, HEATR1, NOLC1, DDX21, GPATCH4, TSEN2, WDR43, PDCD11, MYC, EXOSC2, NPM1, NIFK, UTP4, TEX10, DDX31, WDR12, SNHG29, UCHL5, GNL3, DKC1, NCL, EIF3A* |
| CC | mitochondrion | -3.45 | 1.95E-05 | *TIMM8A, GTF3C4, FASTKD2, TEX10, TWNK, HEATR1, DDX21, MRPL45, TOMM20, LRPPRC, ACACA, HSPD1, AKAP1, CHCHD4, SUPV3L1, MRPL30, YRDC, SLC25A15, MYC, TIMM17A, GUF1, VDAC2, MTPAP, COA7* |
| MF | RNA binding | -4.04 | 1.40E-08 | *UTP25, FASTKD2, WDR3, HEATR1, NOLC1, DDX21, GPATCH4, WDR43, HSPD1, AKAP1, PDCD11, MTPAP, EXOSC2, CCT3, NPM1, NIFK, UTP4, DDX31, MRPL45, PPRC1, LRPPRC, UCHL5, GNL3, SUPV3L1, FUBP3, DKC1, NCL, IMPDH2, EIF3A, FARSB* |
| KEGG | Ribosome biogenesis in eukaryotes | -10.95 | 0.0162 | *UTP4, DKC1, WDR3, HEATR1, WDR43, GNL3* |

Abbreviations: BP: Biological processes; CC: Cellular component; MF: Molecular function

| Upregulated | log2 Fold Change | Adjusted p | Downregulated | log2 Fold Change | Adjusted p |
| --- | --- | --- | --- | --- | --- |
| MS4A1 | 4.618114387 | 5.34E-44 | C1orf68 | -4.382817437 | 0.01084574 |
| FCRL4 | 4.503636157 | 1.39E-26 | SPRR2G | -4.370975951 | 3.04E-06 |
| FCRL1 | 4.449303839 | 7.98E-35 | SPRR2E | -4.289028835 | 2.02E-08 |
| IDO1 | 4.31376282 | 7.75E-85 | KRT9 | -4.03495595 | 1.85E-06 |
| FCER2 | 4.27107745 | 3.16E-28 | LCE3E | -3.915516408 | 0.00454038 |
| IFNG | 4.255619616 | 3.79E-63 | KRT6B | -3.722362694 | 1.27E-09 |
| BANK1 | 4.200323376 | 1.93E-44 | ELSPBP1 | -3.61886567 | 7.45E-05 |
| STAP1 | 4.179115103 | 2.37E-47 | KRT16 | -3.565136758 | 1.40E-06 |
| FCAMR | 4.156164695 | 8.34E-16 | KPRP | -3.497021556 | 0.00191253 |
| FCRL2 | 4.100702598 | 9.82E-41 | SALL3 | -3.491058916 | 0.00917762 |

**Supplementary Table S5**. Top 10 upregulated and downregulated genes in the TNFSF13/CXCL10/CXCL13 high vs. low melanoma patients

**Supplementary Table S6**. Examples of tumor-associated lymphoid aggregates/TLS in MEL PTs with median-high and low levels of serum APRIL/TNFSF13 and CXCL10 or CXCL13.

|  | APRIL/  TNFSF13 | CXCL13 | CXCL10 | Structure/  Immune cell type |
| --- | --- | --- | --- | --- |
| Median (pg/dl) | 584 | 78 | 9 | na |
| Case 1 | 1113 | 77 | 13 | LA CD4+ |
| Case 2 | 1566 | 156 | 11 | LA CD4+ |
| Case 3 | 1260 | 658 | 17 | TLS with GC CD4+ CD20+ |
| Case 4 | 882 | 119 | 21 | TLS CD20+ |
| Case 5 | 803 | 126 | 12 | TLS with GC CD4+CD20+ |

**Supplementary Figure S1.** Serum levels of TLS-kines (CCL19, APRIL, CXCL13, CXCL10) compared between tumors based on presence/absence of tumor-infiltrating lymphocytes. Tumors with brisk/non-brisk tumor-infiltrating lymphocytes (TIL) vs. absence of TIL exhibited significantly higher levels of APRIL/TNFSF13 (p = 0.01), CCL19 (p = 0.01) and CXCL13 (p = 0.01), but no such difference was observed for patients based on serum levels of CXCL10 (p = 0.97).


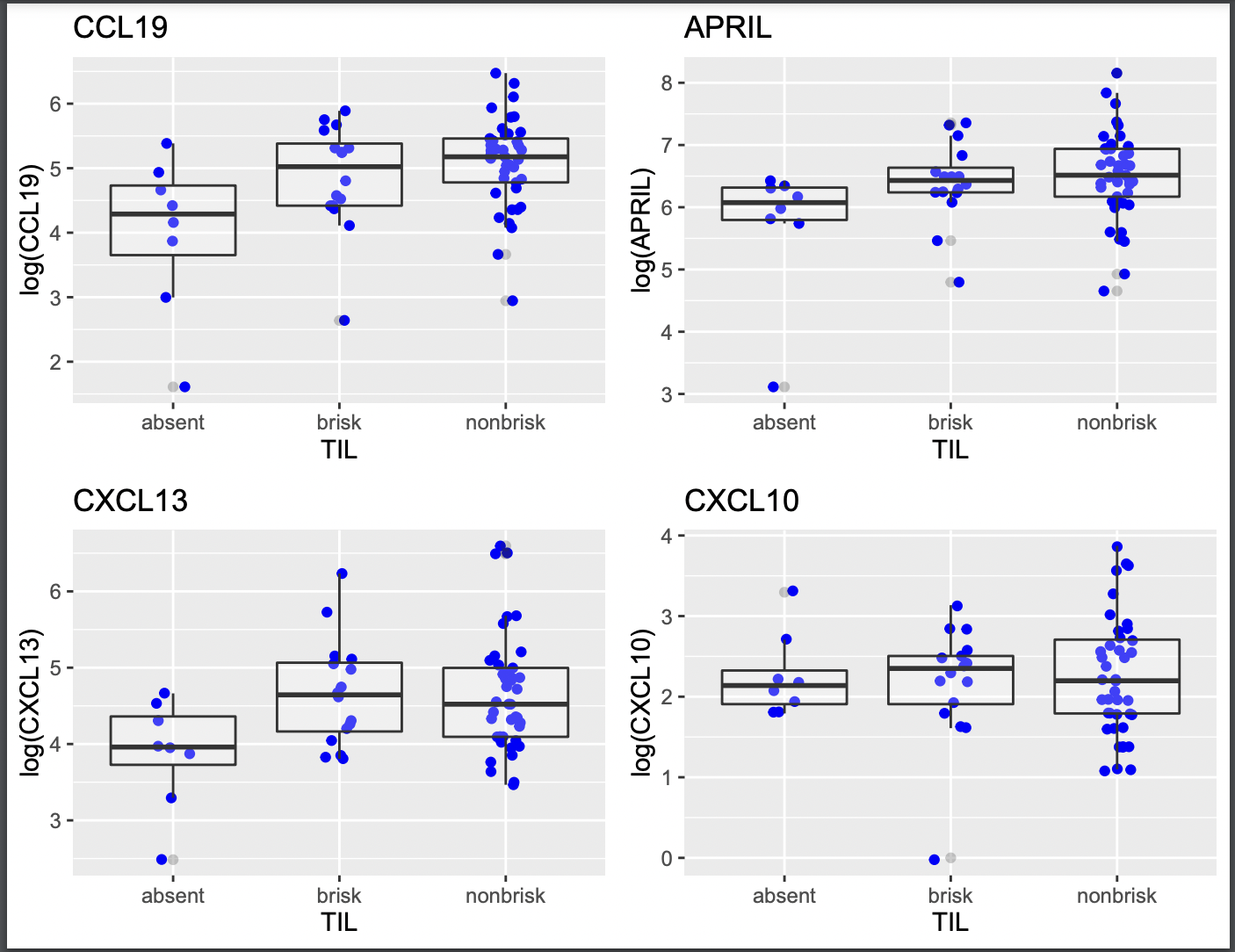


**Supplementary Figure S2. TLS-kine level assessment based on patient melanoma mutational status.** (A) Serum TLS-kines levels are plotted as a function of melanoma driver mutations (BRAF, CDKN2A, NF, NRAS) vs. wild-type (WT) patients. Only CXCL10 levels varied significantly across the different mutation cohorts (p<0.01). (B) TLS-kines mRNA expression levels compared based on the mutational status of *BRAF, NF1, NRAS*. *BRAF* mutated patients had significantly higher expression of *APRIL*/*TNFSF13* and *CXCL10* in comparison to *BRAF* wild-type patients, while *NRAS* wild-type patients had higher expression of *TNFSF13* in comparison to patients with BRAF mutant melanomas.


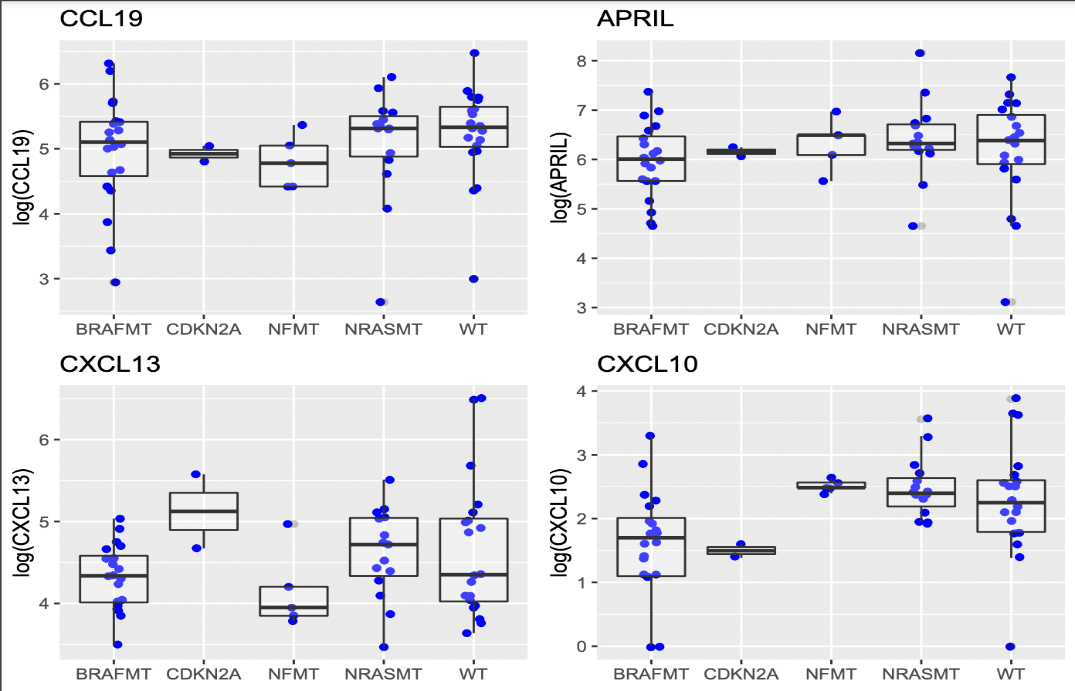


**A**


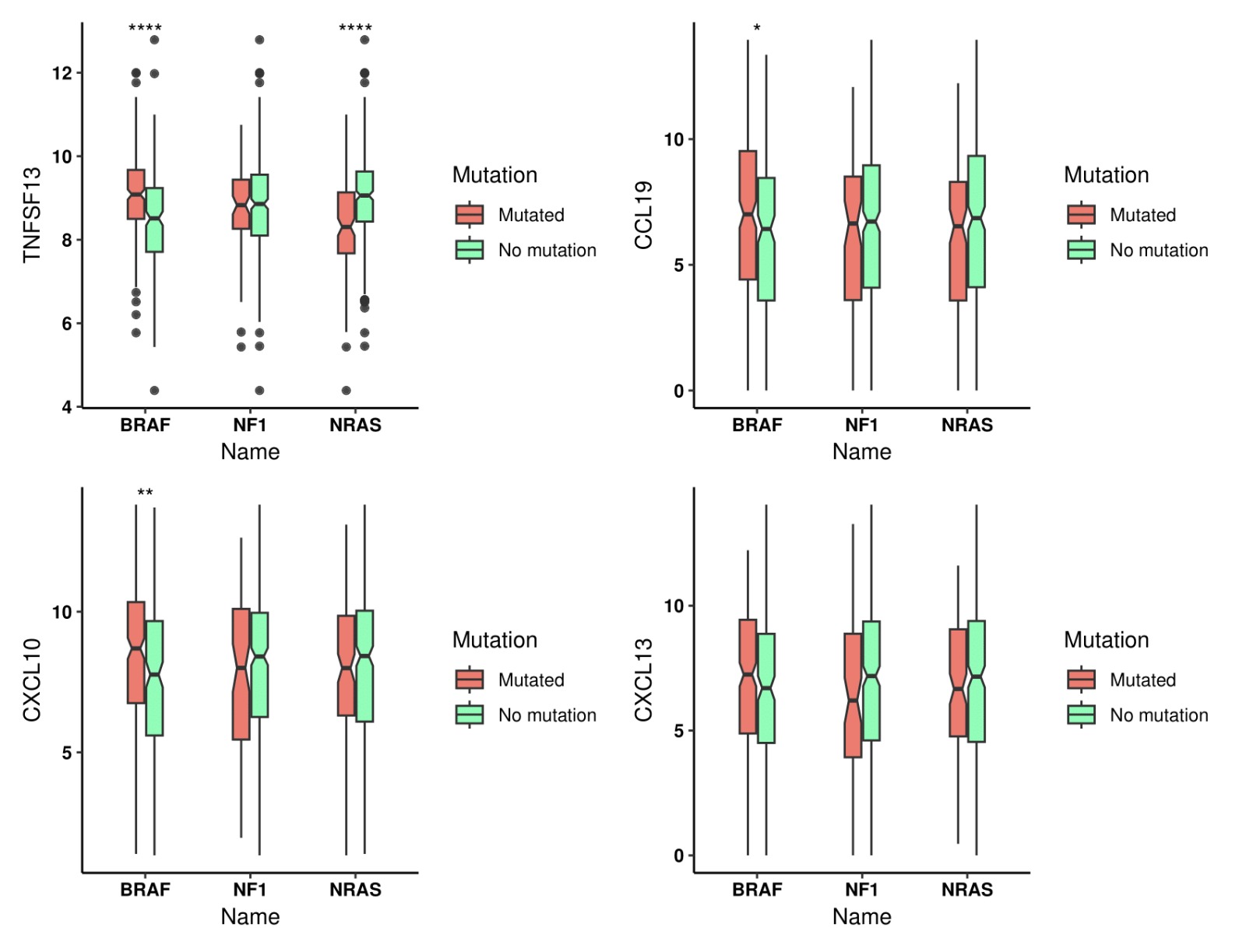


**B**


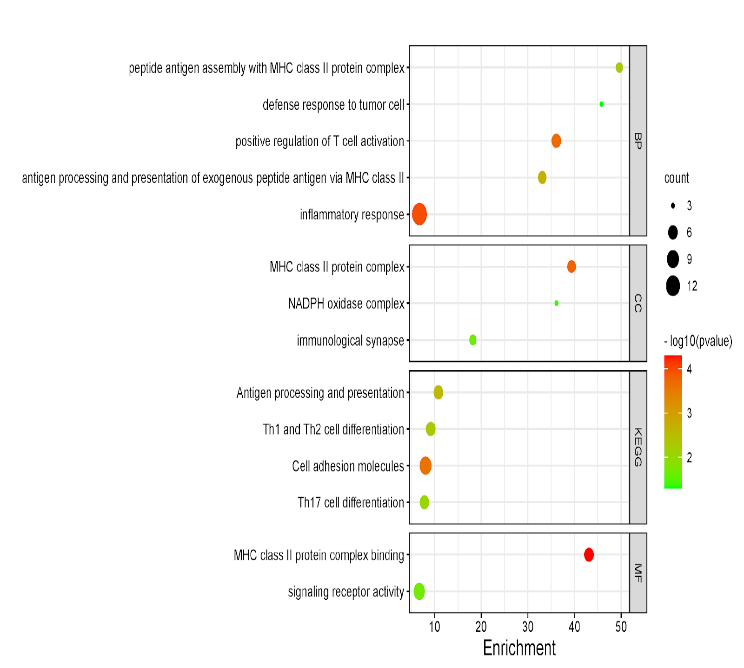


**A**


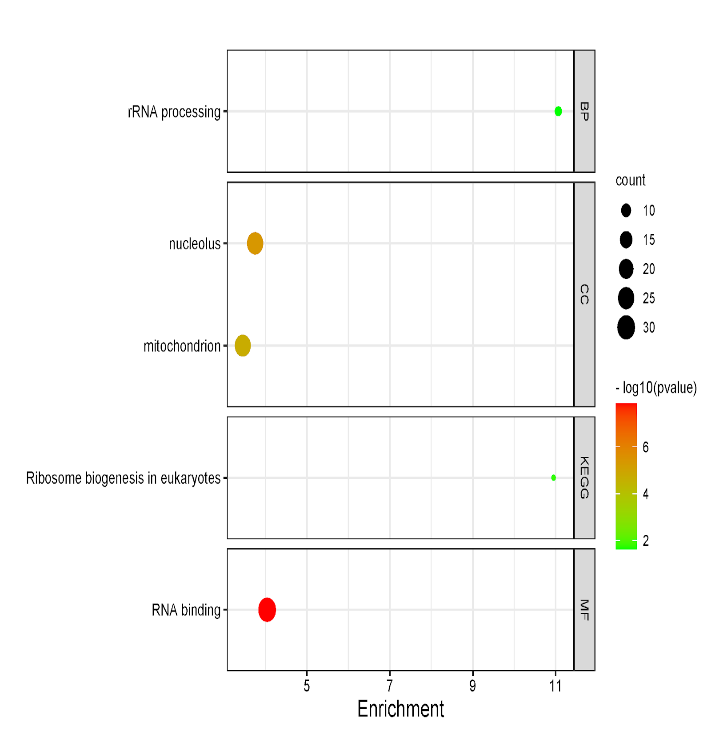


**B**

**Figure S3.** ***APRIL/TNFSF13* associated gene pathways in TCGA melanoma specimens (n = 426).** Bubble plots identifying significantly enriched GO terms for BP, CC, MF and KEGG pathways for (A) positively and (B) negatively co-expressed genes with TNFSF13.


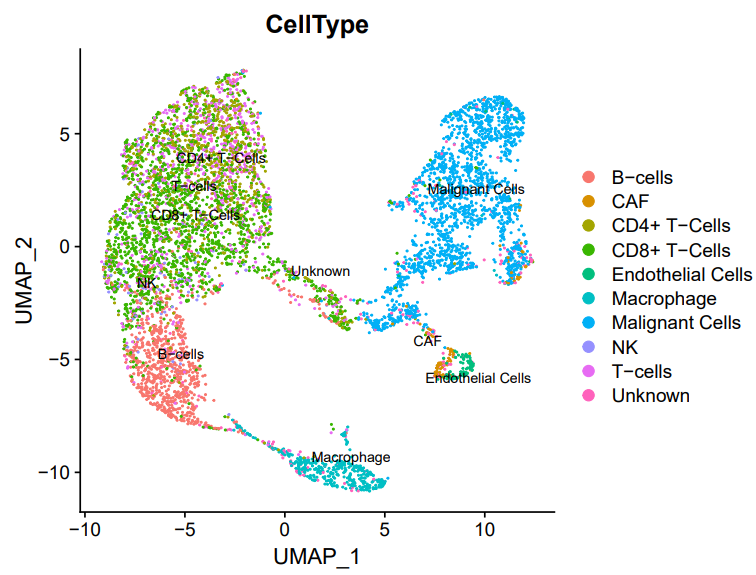


**B**

**A**


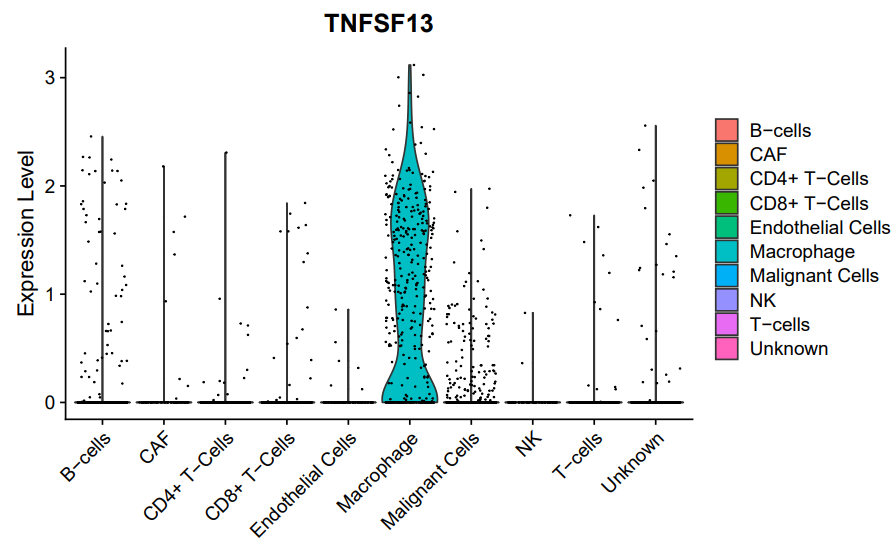


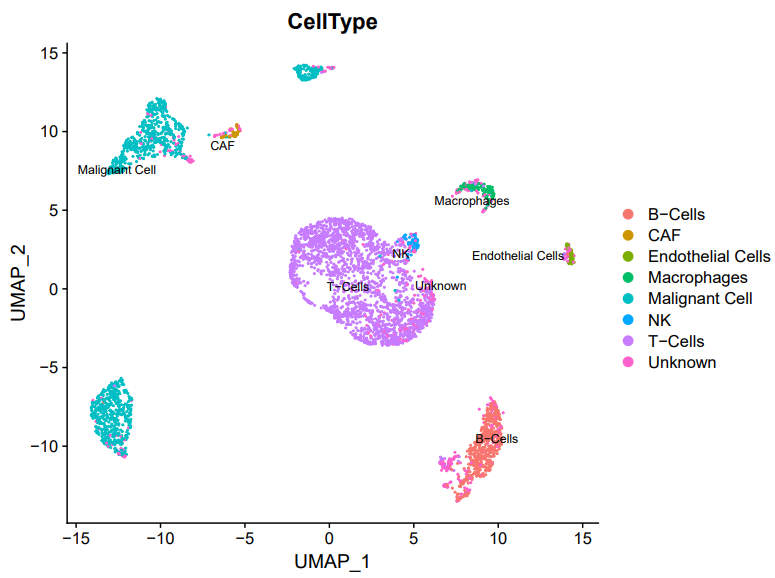

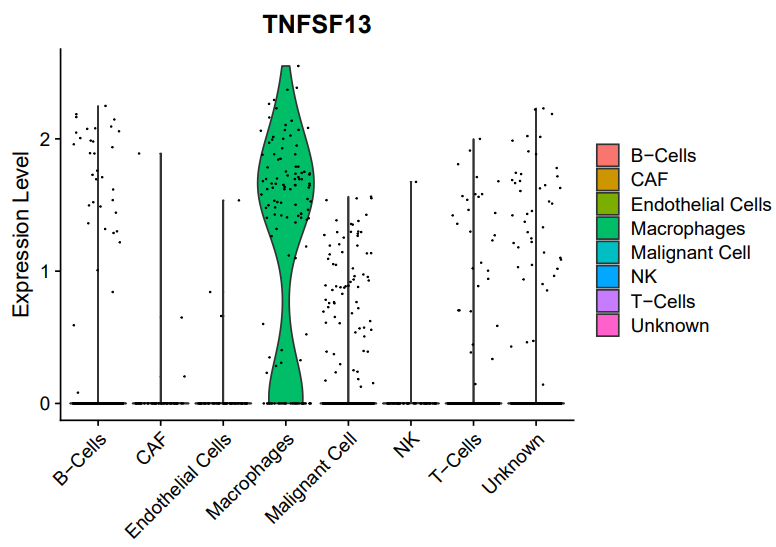


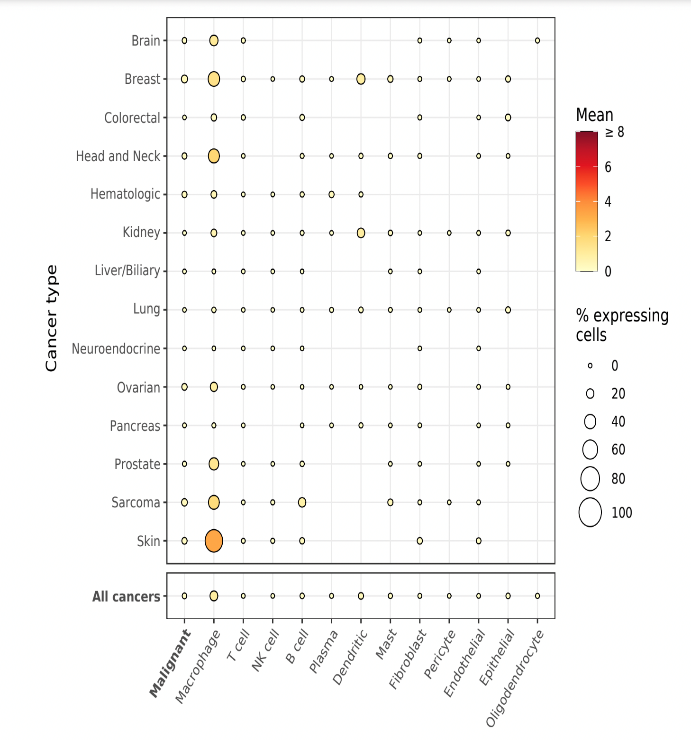


**C**


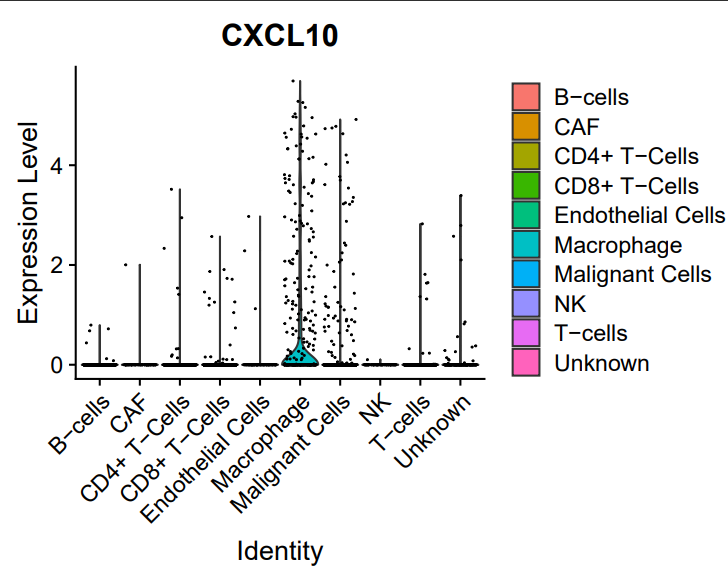


**E**

**D**


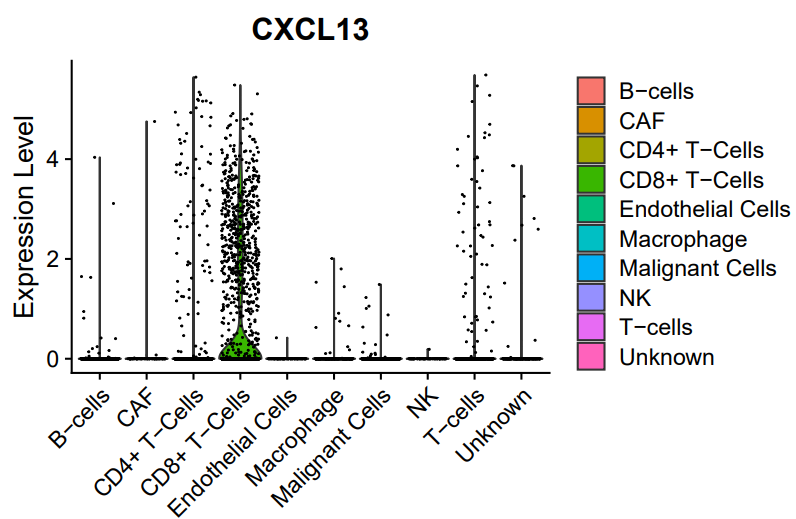


**Figure S4: Single-cell RNA sequence (scRNAseq) analysis of the melanoma TIME using** **GSE115978 and GSE72056. (A)** DimPlot for the UMAP reduction and APRIL/TNFSF13 expression by macrophages in GSE115978 dataset. (**B)** DimPlot for the UMAP reduction and APRIL/TNFSF13 expression by macrophages in GSE72056 dataset. **(C)** Expression of APRIL/TNFSF13 per cell type and cancer type. Plot showing the average expression level of APRIL/TNFSF13 and the percentage of cells expressing APRIL/TNFSF13 in each cancer type and each of the most common cell types, namely those cell types constituting at least 1% of at least 5 individual datasets. Expression levels are defined as log2(TPM/10). Average expression levels and percentages were measured per cell type within each dataset, then averaged across studies within each cancer type. **(D)** CXCL10 expression profiles in single-cell RNA data showing predominant expression by macrophages. (**E)** Violin plot of CXC13 expression showing high expression by CD8+ T-Cells in single-cell data.

**Supplementary Figure S5. IFM-based characteristics of Lymphoid Aggregates/TLS within serum TLS-kine^hi^ MEL PT tumor samples.** (A) Lymphoid aggregates in TLS-kine^hi^ patients (per Supplementary Table S3) show a range of maturity levels including TLS (containing PNAd^+^ and/or CD21^+^ cells) and TLS with GC (containing CD21^+^). (B) Visually apparent lymphoid aggregates were quantified at the whole slide level for each patient and selected for subsequent region of interest analyses. (C) The number of total cells within each lymphoid aggregate was quantified. (D) TLS were defined as a lymphoid aggregate containing PNAd^+^ staining, while TLS + GC were defined as lymphoid aggregates containing CD21^+^ FDC networks. (E) The frequency of Ki67^+^ , CD4^+^ or CD20^+^  cells were quantified across regions after tissue segmentation. N = 5 patients. *p < 0.05 using a Student’s unpaired T test.
